# Systematic Review of the Performance of HIV Viral Load Technologies on Plasma Samples

**DOI:** 10.1371/journal.pone.0085869

**Published:** 2014-02-18

**Authors:** Kimberly A. Sollis, Pieter W. Smit, Susan Fiscus, Nathan Ford, Marco Vitoria, Shaffiq Essajee, David Barnett, Ben Cheng, Suzanne M. Crowe, Thomas Denny, Alan Landay, Wendy Stevens, Vincent Habiyambere, Jos Perrins, Rosanna W. Peeling

**Affiliations:** 1 Department of Clinical Research, London School of Hygiene & Tropical Medicine, London, United Kingdom; 2 Department of Microbiology and Immunology, University of North Carolina, Chapel Hill, North Carolina, United States of America; 3 Department of HIV/AIDS, World Health Organization, Geneva, Switzerland; 4 HIV, Medicine and Science, Clinton Health Access Initiative, New York, New York, United States of America; 5 Department of Haematology, United Kingdom National External Quality Assessment Service (UK NEQAS) for Leucocyte Immunophenotyping, Sheffield, United Kingdom; 6 Department of Technology and Innovation, Pangaea Global AIDS Foundation, San Fransisco, California, United States of America; 7 Centre for Biomedical Research, Burnet Institute, Melbourne, Australia; 8 Department of Medicine, Duke Human Vaccine Institute and Center for HIV/AIDS Vaccine Immunology, Durham, North Carolina, United States of America; 9 Department of Immunology- Microbiology, Rush University Medical Center, Chicago, Illinois, United States of America; 10 Department of Molecular Medicine and Haematology, University of the Witwatersrand, Johannesburg, South Africa; 11 Department of HIV/AIDS, World Health Organization, Geneva, Switzerland; University of Cape Town, South Africa

## Abstract

**Background:**

Viral load (VL) monitoring is the standard of care in developing country settings for detecting HIV treatment failure. Since 2010 the World Health Organization has recommended a phase-in approach to VL monitoring in resource-limited settings. We conducted a systematic review of the accuracy and precision of HIV VL technologies for treatment monitoring.

**Methods and Findings:**

A search of Medline and Embase was conducted for studies evaluating the accuracy or reproducibility of commercially available HIV VL assays. 37 studies were included for review including evaluations of the Amplicor Monitor HIV-1 v1.5 (n = 25), Cobas TaqMan v2.0 (n = 11), Abbott RealTime HIV-1 (n = 23), Versant HIV-1 RNA bDNA 3.0 (n = 15), Versant HIV-1 RNA kPCR 1.0 (n = 2), ExaVir Load v3 (n = 2), and NucliSens EasyQ v2.0 (n = 1). All currently available HIV VL assays are of sufficient sensitivity to detect plasma virus levels at a lower detection limit of 1,000 copies/mL. Bias data comparing the Abbott RealTime HIV-1, TaqMan v2.0 to the Amplicor Monitor v1.5 showed a tendency of the Abbott RealTime HIV-1 to under-estimate results while the TaqMan v2.0 overestimated VL counts. Compared to the Amplicor Monitor v1.5, 2–26% and 9–70% of results from the Versant bDNA 3.0 and Abbott RealTime HIV-1 differed by greater than 0.5log_10_. The average intra and inter-assay variation of the Abbott RealTime HIV-1 were 2.95% (range 2.0–5.1%) and 5.44% (range 1.17–30.00%) across the range of VL counts (2log_10_–7log_10_).

**Conclusions:**

This review found that all currently available HIV VL assays are of sufficient sensitivity to detect plasma VL of 1,000 copies/mL as a threshold to initiate investigations of treatment adherence or possible treatment failure. Sources of variability between VL assays include differences in technology platform, plasma input volume, and ability to detect HIV-1 subtypes. Monitoring of individual patients should be performed on the same technology platform to ensure appropriate interpretation of changes in VL.

Prospero registration # CD42013003603.

## Introduction

As of mid 2013 it is estimated that over nine million HIV infected individuals are on antiretroviral therapy (ART) world-wide and a substantial proportion have been on treatment for ten years or more [1]. As the global ART cohort continues to expand and mature, the need for ongoing monitoring is becoming increasingly important to ensure treatment efficacy and minimize the risk of HIV drug resistance. Clinical and immunological monitoring techniques have poor sensitivity and specificity for detecting virologic failure, leading to a substantial misclassification of treatment responses, resulting in delayed switching in some cases and inappropriate switching from first line regimens in others [2]–[7].

Routine HIV viral load (VL) monitoring has the potential to improve the accuracy of diagnosis of treatment failure, enable more targeted adherence interventions, and preserve the efficacy of ART [8]. Monitoring HIV VL is often not performed in resource-limited settings because the assays are costly, and require sophisticated, expensive laboratory equipment and trained technicians [9], [10]. Despite these limitations, the importance of HIV VL testing is increasingly recognized: in 2010 the World Health Organization (WHO) recommended that countries begin to phase in VL for monitoring patients on ART [1], a recommendation reinforced in the 2013 treatment guidelines [11]. Detailed descriptions of available VL technologies can be found in a UNITAID HIV/AIDS diagnostic landscaping [12].

In order to support decisions regarding which VL tools to phase in, we conducted a systematic review of the performance and operational characteristics of commercially available HIV VL assays.

## Methods

We first verified that no systematic reviews had already been conducted on this topic by searching the Cochrane Library and Centre for Reviews and Dissemination, University of York and National Institute for Health Research. A research protocol was then developed following standard guidance [13] and this was reviewed by all members of the HIV Monitoring Technologies Working Group before the search was performed. The systematic review protocol was registered with PROSPERO (http://www.crd.york.ac.uk/PROSPERO), registration number CD42013003603.

### Search

Medline and Embase were searched using the search terms (‘HIV-1’ or ‘HIV-2’ or ‘HIV’ or ‘human immunodeficiency virus’ or ‘HIV type 1’ or ‘HIV type 2’ or ‘human immunodeficiency virus type 1’ or ‘human immunodeficiency virus type 2’) and (‘viral load’ or ‘viral RNA’) and (‘compar*’ or ‘eval*’) and (‘measur*’ or ‘quant*’ or ‘technol*’ or ‘test’) and (‘accuracy’ or ‘performance’ or ‘precision’ or ‘sensitivity’ or ‘specificity’ or ‘sensitivity and specificity’). Results of the search were exported to EndNote X3, duplicates removed and the remainder assessed for relevance and fulfillment of the selection criteria.

### Study Selection

The search was conducted in February 2010 and updated in April 2012 to include scientific research articles published in peer-reviewed journals, in English, between January 1990 and the search date. Publications evaluating or comparing the performance of commercial assays for the quantification of HIV-1 or HIV-2 virus load in plasma were included in the search.

There were no limitations on the method of nucleic acid extraction, amplification, or detection but the assays under investigation had to be commercially available at the time of the review. The study population was limited to adults but no restriction was placed on the geographical origin of the samples or the HIV subtype (HIV-1 or HIV-2). Publications using samples from standardized panels were also considered for inclusion providing they met the study criteria. No authors were contacted for further information and all data presented in this review were available in the included publications.

### Data collection processes

Two independent reviewers extracted data on assay accuracy and reproducibility from publications meeting the inclusion criteria as defined in the protocol. Where there was any discrepancy, the reviewers met to discuss the difference and came to a consensus on inclusion or exclusion from the study. The quality of publications included in the HIV VL review was scored using adapted STARD guidelines [14], [15]. This included questions on the title and abstract; introduction; methods including participant/sample characteristics, test methods and statistical methods; results including data on participants and test results; and discussion (Annex S3). The two reviewers selected 17 critical quality criteria of the original 23 which were more appropriate for evaluations of quantitative assays.

### Quantitative data synthesis

Accuracy and reproducibility data were summarized graphically in Excel. Accuracy measures included bias and limits of agreement [16], sensitivity and specificity, and the percentage of results differing by 0.5Log_10_, which is generally considered the clinically relevant difference between two VL measurements [17], [18]. Reproducibility measures included within- and between-assay variability, reported as % coefficient of variation (%CV).

## Results

### Study selection

**Figure 1:**
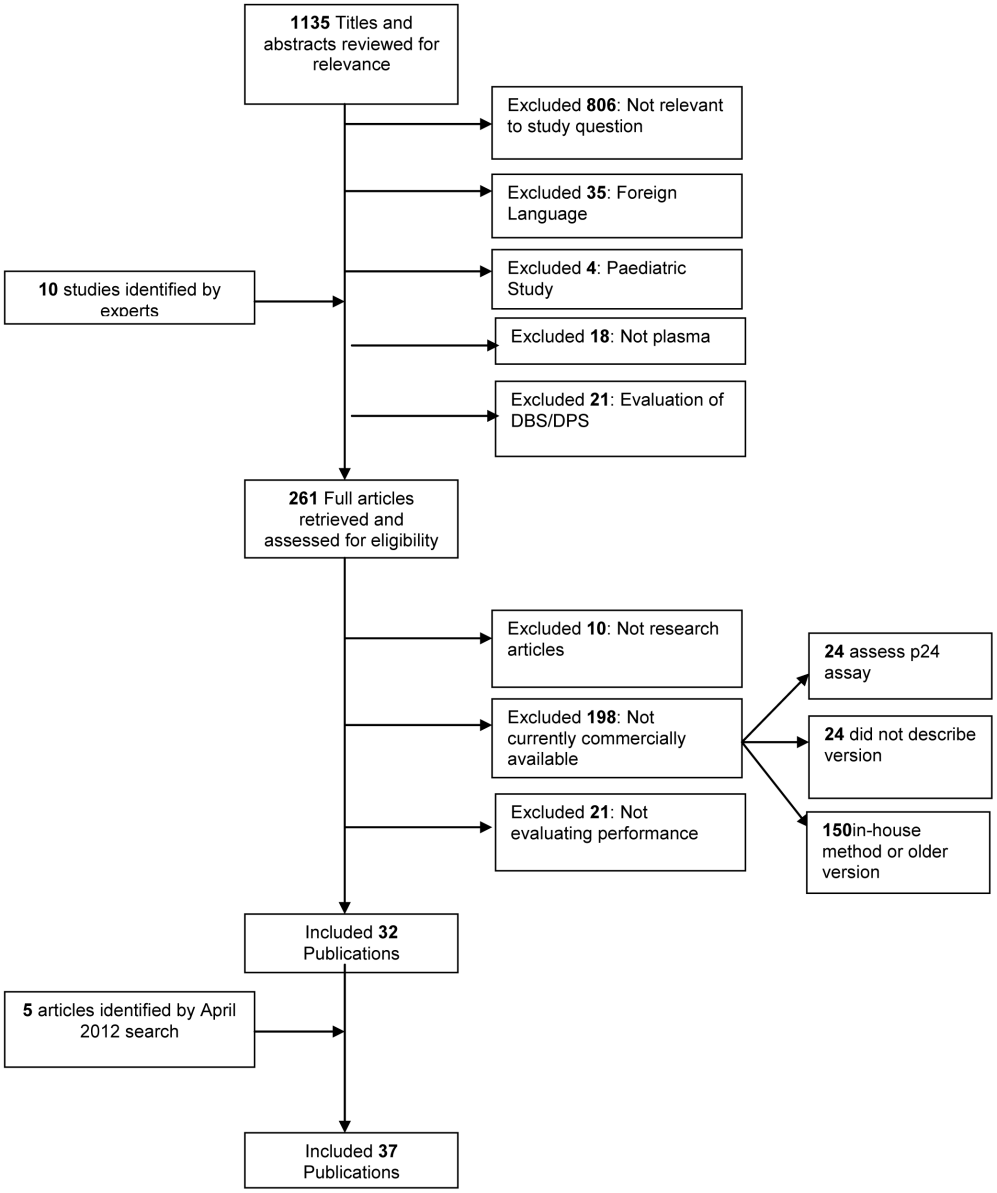
HIV VL Search Algorithm.

The search produced 1,715 titles, of which 580 were removed as duplicates. Of the remaining 1,135 titles and abstracts, 261 publications were reviewed as full text, and 37 met the criteria for inclusion and were taken forward for inclusion in the review (Figure 1) [19]–[55].

### Study characteristics

The studies included data on the following assays: Roche Amplicor Monitor v1.5 (Amplicor 1.5) (n = 25), Roche Cobas TaqMan v2.0 (TaqMan v2.0) (n = 11), Abbott RealTime HIV-1 (Abbott RealTime) (n = 23), Versant HIV-1 RNA bDNA 3.0 (bDNA 3.0) (n = 15), Versant HIV-1 RNA kPCR 1.0 (kPCR 1.0) (n = 2), Cavidi ExaVir Load v3 (ExaVir v3) (n = 2) and the NucliSens EasyQ 2.0 (EasyQ 2.0) (n = 1). Only one publication evaluated assays using HIV-2 samples (29) and the remainder used HIV-1 Group M, N, and O. There was no single standard reference test used as a comparator in all studies. Of the 37 included studies, 12 were published in 2009 (median), with a minimum and maximum of 2000 and 2012 respectively.

### Quantitative Data Synthesis: Accuracy of HIV VL Assays

#### Analytical Sensitivity and Specificity of HIV VL Assays with Plasma

One study provided analytical sensitivity and specificity data for ExaVir Load v3 compared to Amplicor Monitor v1.5 [28] (Table 1). Sensitivity of the ExaVir v3 was 96–100% at HIV VL concentrations above 2000 copies/mL, but decreases to 59% when VL concentration decreased to between 50–400 copies/mL. The specificity of the ExaVir v3 was evaluated using HIV-1 negative samples and reported as 100% [26].

**Table 1:** Analytical Sensitivity of the ExaVir Load v3 compared to Amplicor Monitor v1.5.

Cut-off (copies/mL)	Publication	N	Sensitivity
>200	(28)	199	92%
>400	(28)	178	94%
>1000	(28)	145	98%
>5000	(28)	23	100%
50–400	(28)	64	59%
401–2000	(28)	57	86%
2001–10 000	(28)	50	96%
10 001–50 000	(28)	48	100%

Five studies evaluated the specificity of the Amplicor v1.5, Abbott RealTime, bDNA 3.0, and ExaVir v3 assays using HIV-1 negative samples and reported as 100% [15], [18], [28], [45], [49]. When tested with a panel containing four HIV-2, four HCV, and four polyomavirus BK plasma samples, the specificity of the kPCR was 92% [39]. One study evaluated the specificity of the TaqMan v2.0 using HIV-1 negative samples containing potentially cross-reactive reactive viruses (including adenovirus Type 5, cytomegalovirus, Epstein-Barr virus, hepatitis B, C, and A viruses, herpes simplex virus Type I and Type II and others (n = 660) and found no false positive results or cross-reactivity [44].

#### Clinically important differences in result

Seventeen studies evaluated the percentage of results differing by a clinically important value, defined as 0.5log_10_, between the index test and reference test [22], [27]–[29], [31], [38]–[41], [45], [48]–[51], [55]. Data were available for Amplicor v1.5, TaqMan v2, Abbott RealTime, bDNA 3.0, kPCR, and ExaVir v3.

**Figure 2:**
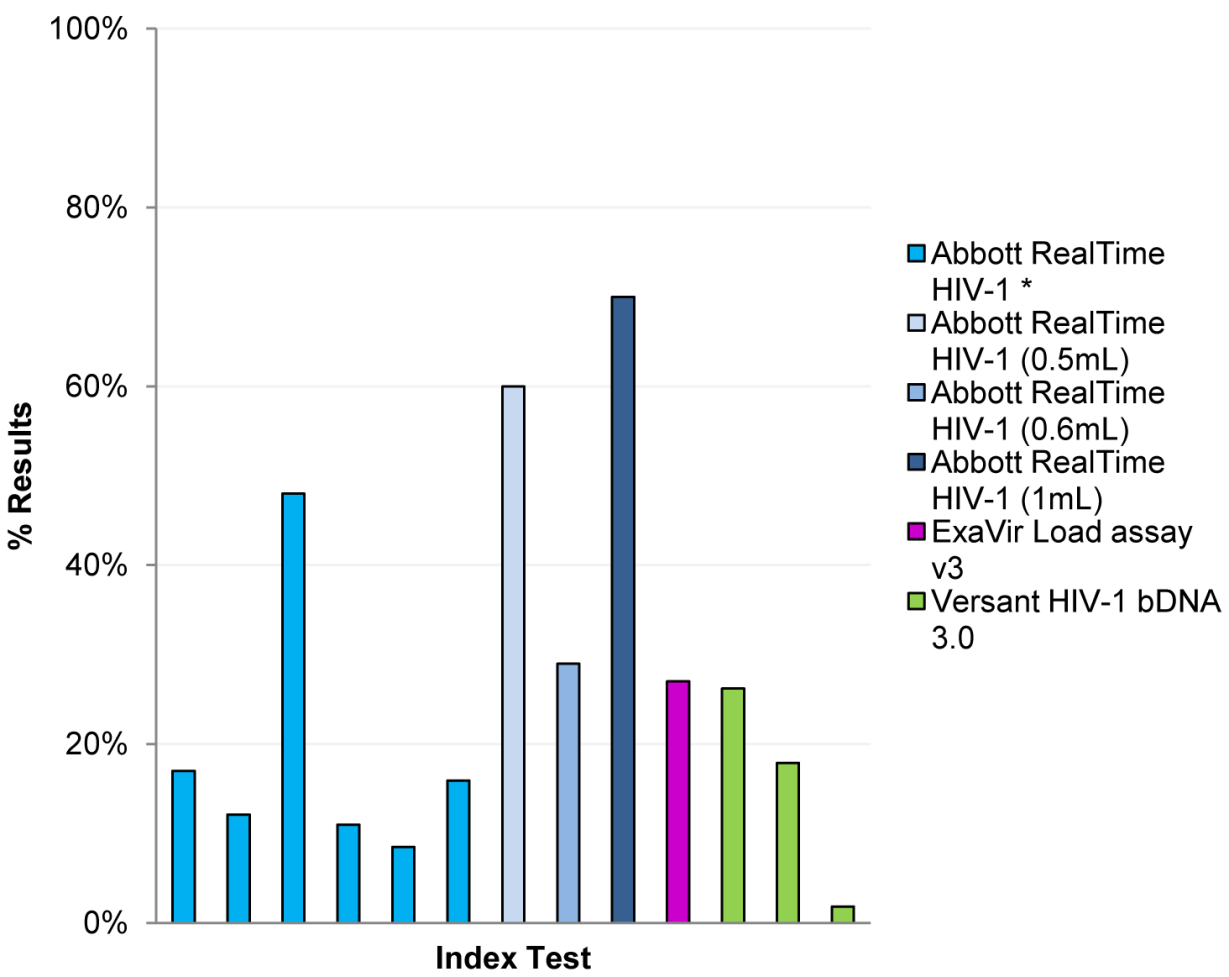
% of results differing by >0.5log10 between index test and Amplicor v1.5 (data extracted from references [22], [27]–[29], [38], [40], [45], [48], [49], [55]). *No volume specified.

Between 8.5% [49] and 70.0% [38] of results provided by the Abbott RealTime assay differed by greater than 0.5log_10_ compared to the Roche Monitor v1.5 (Figure 2). The greatest differences in results occurred using the 1 mL Abbott RealTime sample input, where 70% of results differed by more than 0.5log_10_ compared to the Amplicor 1.5 [38]. Results from the bDNA 3.0 showed much lower levels of discordance compared to the Amplicor 1.5, with between 2–26% of results having clinically important differences [22], [27]. Only one study reported differences between results from the ExaVir v3.0 and the Amplicor v1.5; in this study, 27% of results from the ExaVir differed by more than 0.5log_10_ [28].

When comparing the assays to the Abbott RealTime assy, between 10 and 30% of TaqMan results [41], [51], and 13% of bDNA v3 results [48] differed by more than 0.5log10.

#### Bias

**Figure 3:**
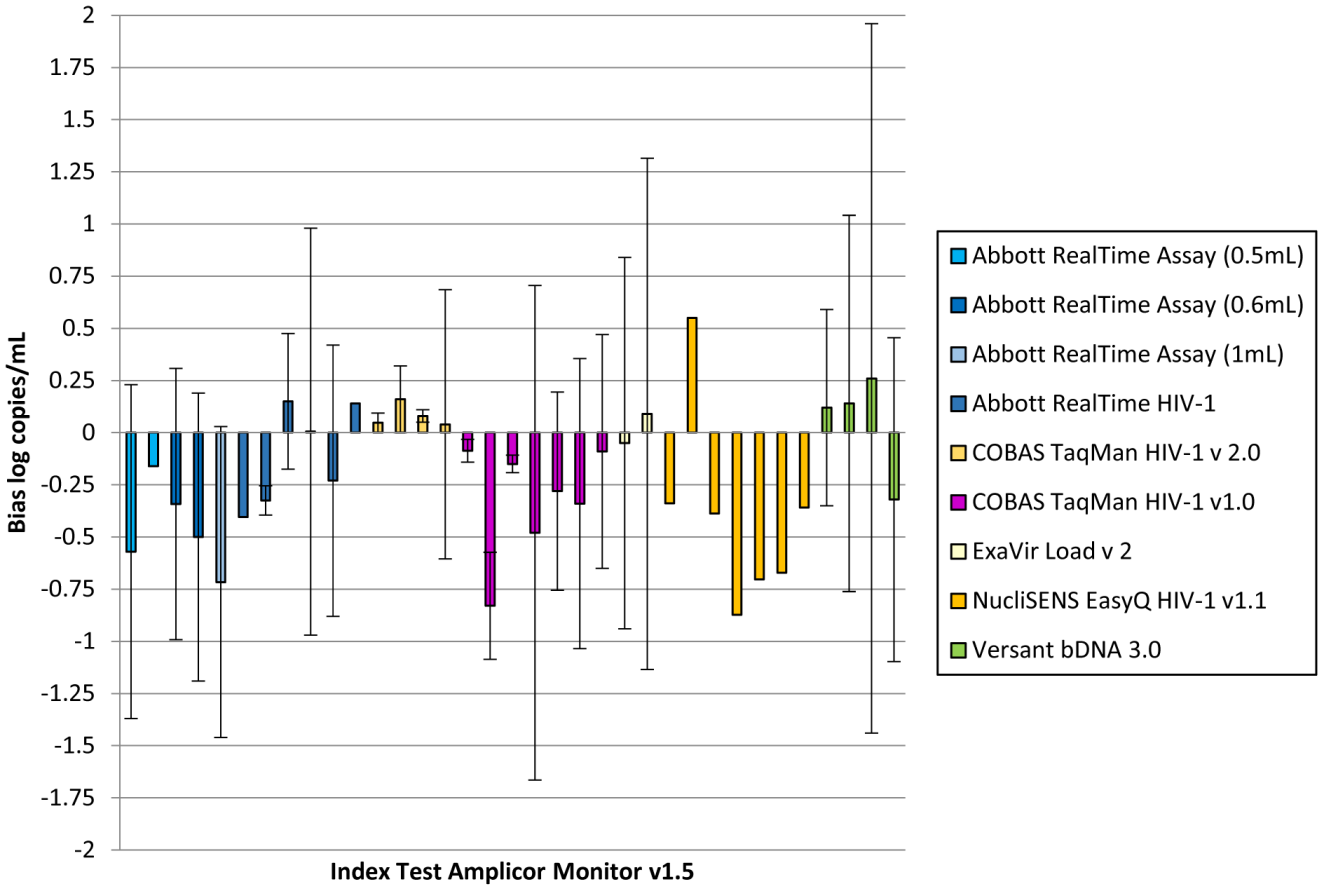
Bias between Index Test and Ampicor v1.5 as a comparator (data extracted from references [19], [24], [25], [29], [37], [38], [40], [41], [43], [45], [46], [48], [52], [55]). *No volume specified.

Bias was measured between an index test and the Amplicor v1.5 as a reference standard in 14 publications [19], [24], [25], [29], [37], [38], [40], [41], [43], [45], [46], [48], [52], [55]. These included comparisons with the Abbott RealTime (n = 11) [19], [25], [29], [38], [40], [45], [46], [48], [55], TaqMan v2.0 (n = 4) [24], [25], [41], and bDNA 3.0 (n = 4) [37], [43], [52]. There were no bias data available comparing the ExaVir v3, EasyQ v2.0, or the kPCR to the Monitor v1.5. Figure 3 summarizes the range of bias measurements reported and the variability in the limits of agreement reported for each assay when evaluated alongside the Amplicor v1.5.

The EasyQ reports results as IU/mL (International Units/mL). A conversion factor supplied by the manufacturer was applied to enable comparison with other studies; however this process did not produce consistent results when applied to the limits of agreement.

**Figure 4:**
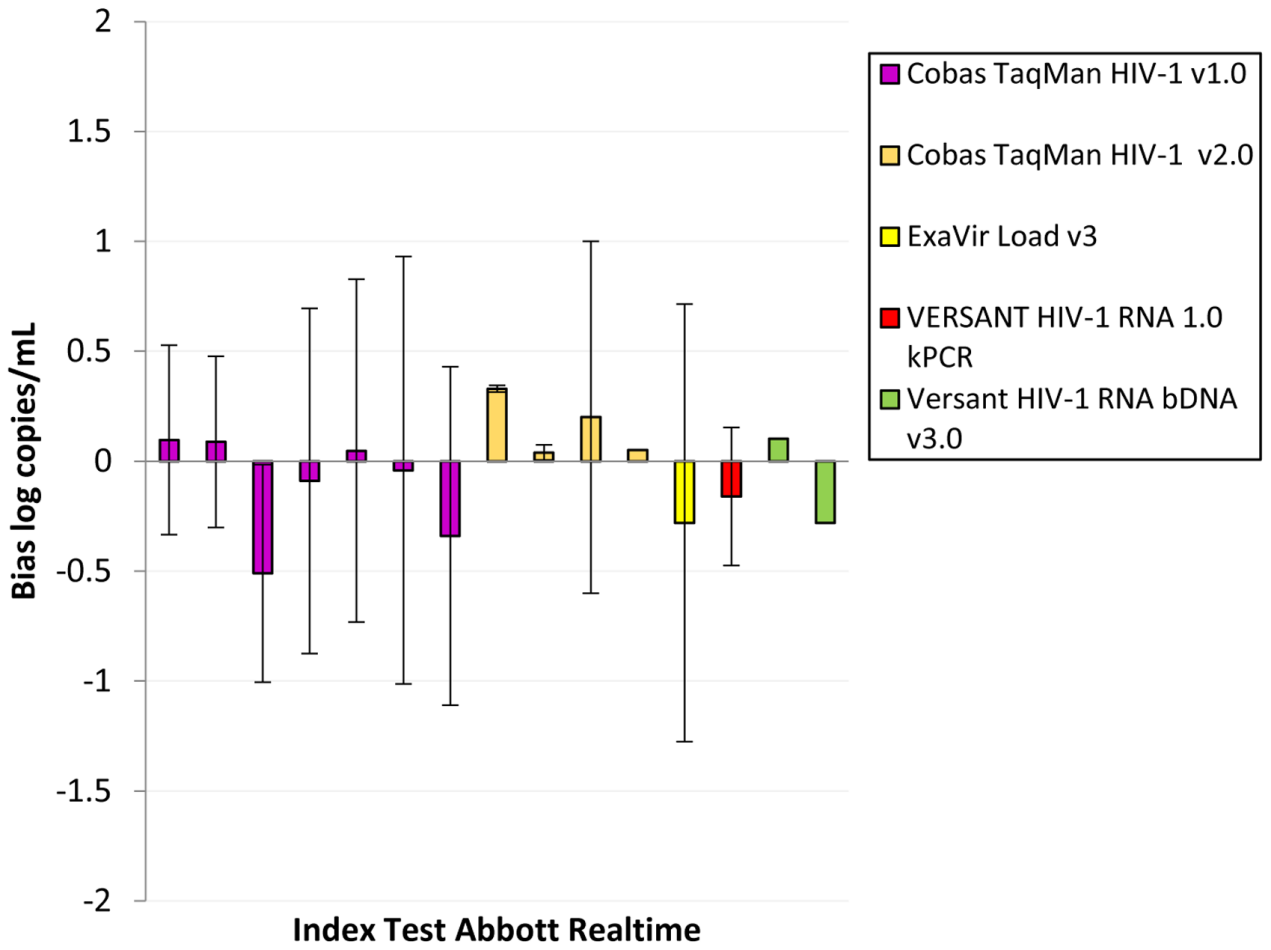
Bias between Index Test and Abbott RealTime as a comparator (data extracted from references [19], [30], [32], [35], [39], [41], [42], [48]).

**Figure 5:**
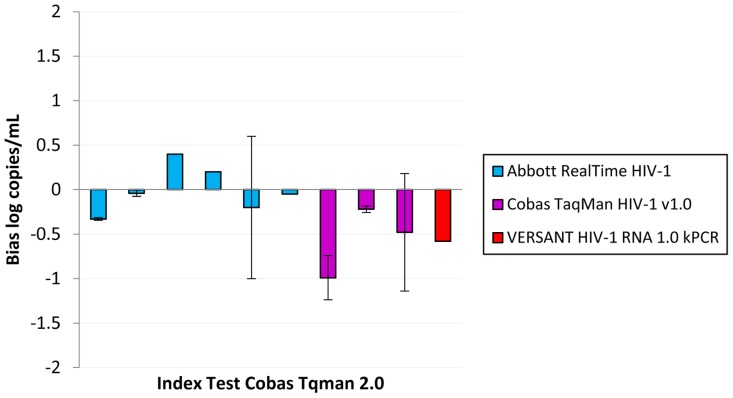
Bias between Index Test and TaqMan v2.0 as a comparator (data extracted from references [30], [35], [41], [42], [50], [51], [54]). *No volume specified.

Data on bias were also available comparing index tests to the Abbott RealTime (Figure 4) [19], [29], [30], [32], [35], [39], [41], [42], [45], [48], [53], TaqMan v2.0 (Figure 5) [24], [30], [35], [41], [42], [50], [51], [54] and to a WHO International Standard (Table 2) [51]. Compared to the Abbott RealTime, the Taqman v2.0 overestimated VL counts by 0.04–0.33 log_10_ c/mL [30], [35], [41], [42], while the ExaVir v3 and kPCR underestimated VL but only by 0.28 and 0.16 log_10_c/mL, respectively [32], [39]. The limits of agreement associated with the ExaVir v3 spanned from −1.27 to 0.72 log_10_c/mL [32], and those associated with the kPCR data point from −0.474 to 0.154 log_10_c/mL [39]. When index tests were compared to the TaqMan v2.0, the kPCR underestimated VL [50]. The Abbott RealTime both over and under-estimated HIV VL values but never by more than 0.5log_10_ [30], [35], [41], [42], [51]), with the exception of one study that reported limits of agreement spanning from −1 to 0.6 log_10_ c/mL [42]. EasyQ 2.0 overestimated VL by 0.88log_10_ c/mL [54]. Finally, when compared to the WHO International Standard, neither the Abbott RealTime nor the TaqMan v2.0 differed by more than 0.5log_10_ with the Abbott RealTime displaying slight overestimation while the TaqMan v2.0 showed slight underestimation of HIV VL [51]).

**Table 2:** Bias data between index test and WHO International Standard [50].

Index Test	Reference Standard (mean log copies/ml)	Bias	Limits of Agreement (min)	Limits Of Agreement (max)
Abbott RealTime HIV-1	WHO International Standard Low (2.29)	−0.3	−0.44	−0.16
Abbott RealTime HIV-1	WHO International Standard High (4.38)	−0.26	−0.31	−0.21
Roche TaqMan v2.0	WHO International Standard Low (2.30)	0.31	0.22	0.39
Roche TaqMan v2.0	WHO International Standard High (4.40)	0.17	0.1	0.23

### Quantitative Data Synthesis: Precision of HIV VL Assays

**Figure 6:**
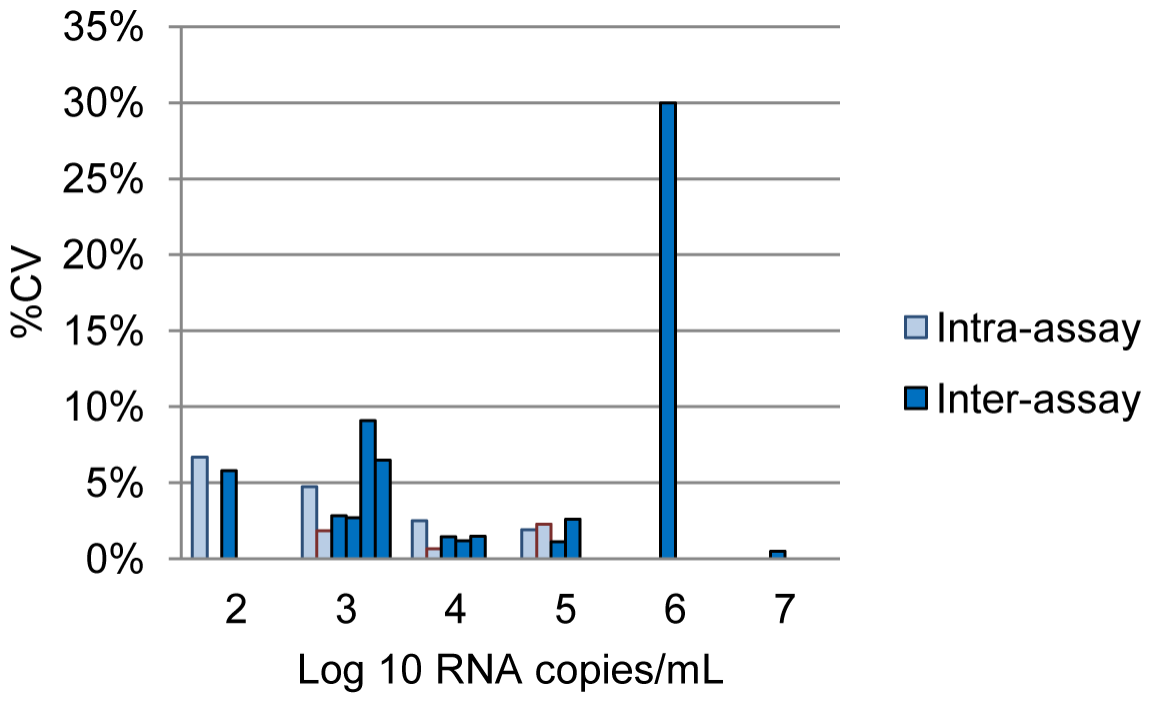
Intra- and inter-assay variation for the Abbott RealTime HIV-1(plasma) according to log copy number/mL of sample (data extracted from references [19], [20], [23], [38], [40], [45]).

**Figure 7:**
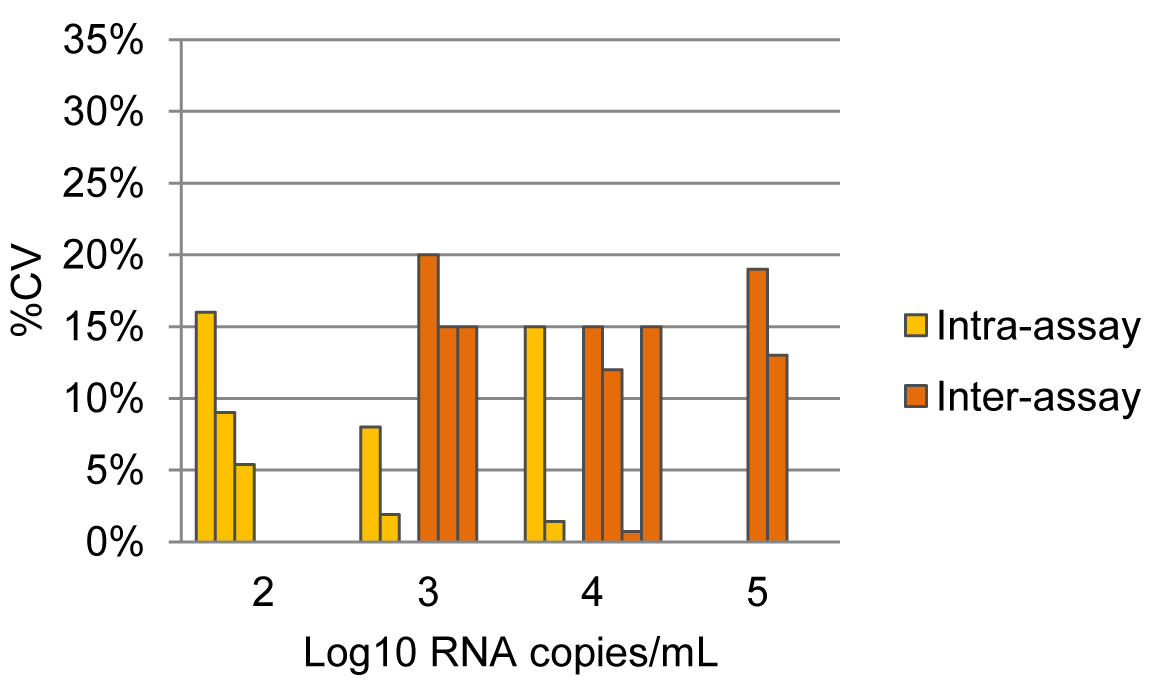
Intra- and inter-assay variation for the Versant kPCR (plasma) according to log copy number/mL of sample (data extracted from references [39], [50]).

Data on intra-assay (within-run) and inter-assay (between-run) variability were reported for six assays: Amplicor v1.5 [23], [47], Abbott RealTime (Figure 6) [19], [20], [23], [38], [40], [45], and the kPCR (Figure 7) [39], [50]. The Abbott RealTime showed excellent intra- and inter-assay reproducibility (<10% variability) at low copy numbers. One study, however, found the inter-assay variability to be 30% at 500 000 c/mL [19]. The kPCR showed overall intra- and inter-assay variability exceeding 15% [39], [50].

### Quality Assessment of Studies Included in the HIV VL Review

All thirty-seven articles included in the review were assessed for quality by two independent reviewers (Annex S1, S2). No article met all 17 quality assessment criteria. The quality scores ranged from 24–94%, and the median was 65%. While 95% of articles described the study aims, only 8% reported on staff training. Twenty-three (62%) and twenty-six (70%) of included publications clearly described sample acquisition and sample storage conditions, respectively. Twenty-one studies (57%) detailed the statistics performed but only 16 (43%) presented descriptive statistics and bias calculations. All studies discussed the clinical relevance of their findings.

## Discussion

In 2013, the World Health Organization recommended that, with the exception of dried blood spot samples, the threshold for detection of virological failure should be lowered to 1000 c/mL. This recommendation was made in support of a shift towards a more strategic use of antiretrovirals both for the treatment of HIV infection and also the prevention of onward transmission through earlier initiation of ART among priority groups such as pregnant women and serodiscordant couples [11].

This review found that all the assays currently in use can reliably detect HIV VL of 1000 c/mL, which is within the linear ranges of VL assays claimed by manufacturers (Table 3). If a threshold of ≥1000 c/mL is used to consider switching to a second line regimen, then all assays were found to have acceptable performance to be of use in clinical decision making. The challenge of routinely and reliably detecting 1000 c/mL may be of greater concern for the next generation of point-of-care tests.

**Table 3:** Manufacturer Data: Plasma Input Volume and Linear Range of Detection.

Manufacturer: Assay	Input vol	Linear Range (c/ml)
Abbott RealTime HIV-1	0.2 mL	0.2 mL sample input: 150–10,000,000
	0.5 mL	0.5 mL sample input: 75–10,000,000
	0.6 mL	0.6 mL sample input: 40–10,000,000
	1.0 mL	1.0 mL sample input:40–10,000,000
bioMérieux NucliSens EasyQ HIV-1 v2.0	0.1 mL	10–10,000,000
Cavidi ExaVir Load	1 mL	200–600,000
Roche Diagnostics Cobas Amplicor HIV-1 MONITOR TEST, v1.5	0.5 mL	Standard protocol: 400–750,000
		Ultra-sensitive protocol: 50–100,000
Roche COBAS AmpliPre/COBAS TaqMan HIV-1 Test, v2.0	1 mL	20–100,000,000
Siemens VERSANT HIV-1 RNA 3.0 assay (bDNA)	1.0 mL	50–500,000
Siemens VERSANT HIV-1 RNA 1.0 assay (kPCR)	0.65 mL	37–11,000,000

The most difficult aspect of conducting this review was the different reference standards used for evaluating test performance in different studies. For comparability it would be useful if a single standard measurement was used for HIV VL. The NucliSens EasyQ is the only assay to report results using IU/mL. As technologies evolve, a consensus international standard for HIV VL copies that is widely accessible would provide a valid and easier reference standard for determining the analytical performance of a new assay.

Sources of variability between VL assays reported include not only differences in technology platform, but also plasma input volume, and ability to detect HIV-1 subtypes. VL monitoring should therefore be performed on the same technology platform for monitoring individual patients to ensure appropriate interpretation of changes in VL, unless clinically relevant differences are not identified between different assays.

Interpretation of the data available was also limited by the variable quality of the publications. This review highlights the need for more rigor in the design and reporting of evaluations of HIV VL quantification technologies, particularly as new versions of HIV VL assays and point-of-care (POC) formats become available. One shortcoming highlighted by the review is the incorrect application of statistical techniques. Correlation and linear regression were the most common measurements reported but bias and limits of agreement would be much more informative. Unlike linear regression, Bland-Altman plots describe the mean difference between two sets of data points and give this value a direction indicating whether the index test is likely to under- or over-quantify results [16]. As with CD4 quantification technologies, the extent of misclassification above and below a clinically important threshold will need to be investigated [56]. It is important for future studies to report the frequency, intensity and direction of the misclassifications [1], because misclassification can have clinical and public health implications (patients are left on failing regimens and may develop drug resistance) and economic implications (second line regimens are often expensive and options beyond second-line are limited). Precision or reproducibility should also be detailed with a clear description of how the measures were obtained, including information on number of samples, number of replicates per sample, and a descriptive summary of the characteristics of the samples used including mean HIV VL (± SD) and range. The results of the review show that standardized practices and guidelines for improved methods undertaking and reporting evaluations of HIV VL assay evaluations are needed, particularly with respect to defining the study population, reporting algorithms for inclusion and exclusion of samples throughout the study, reporting training of technicians, and the use of appropriate statistical methods [11].

The main limitation of this review methodology is that the inclusion criteria were limited to studies published in English, which may have overlooked useful data available in other languages.

Since the results of our review indicate that all currently commercially available HIV VL assays can provide a reliably accurate measure of plasma VL ≥1000 c/mL, switching from the current WHO recommended threshold of 5,000 c/mL for investigations for treatment compliance or possible treatment failure to 1,000 c/mL would allow earlier detection of treatment failure, enable more targeted adherence interventions, and preserve the efficacy of ART. Choice of technology platform should take into account the ability to detect HIV-1 subtypes in the target population. Serial samples for VL monitoring need to be performed on the same technology platform for proper interpretation of any meaningful changes in VL.

## Supporting Information

Annex S1 **Quality assessment results by 17 criteria.**
(TIF)

Annex S2 **Review Protocol.**
(DOCX)

Annex S3 **PRISMA Guidelines.**
(DOC)

## References

[1] World Health Organization (2010) Antiretroviral Therapy For HIV Infection in Adults and Adolescents Recommendations for a public health approach: 2010 revision. Geneva.: World Health Organization. 23741771

[2] Bisson GP, Gross R, Strom JB, Rollins C, Bellamy S, et al (2006) Diagnostic accuracy of CD4 cell count increase for virologic response after initiating highly active antiretroviral therapy. AIDS Patient Care STDS 20 12: 1613–1619. 10.1097/01.aids.0000238407.00874.dc 16868442

[3] Mee P, Fielding KL, Charalambous S, Churchyard GJ, Grant AD (2008) Evaluation of the WHO criteria for antiretroviral treatment failure among adults in South Africa. AIDS 22: 1971–1977. 18784460 10.1097/QAD.0b013e32830e4cd8

[4] Mellors JW, Munoz A, Giorgi JV, Margolick JB, Tassoni CJ, et al (1997) Plasma Viral Load and CD4+ Lymphocytes as Prognostic Markers of HIV-1 Infection. Ann Intern Med 126: 946–954. 9182471 10.7326/0003-4819-126-12-199706150-00003

[5] Moore DM, Awor A, Downing R, Kaplan J, Montaner JSG, et al (2008) CD4+ T-Cell Count Monitoring Does Not Accurately Identify HIV-Infected Adults With Virologic Failure Receiving Antiretroviral Therapy. J Acquir Immune Defic Syndr 49 5: 477–484. 18989232 10.1097/QAI.0b013e318186eb18

[6] O'Brien WA, Hartigan PM, Daar ES, Simberkoff MS, Hamilton JD, et al (1997) Changes in Plasma HIV RNA Levels and CD4+ Lymphocyte Counts Predict Both Response to Antiretroviral Therapy and Therapeutic Failure. Ann Intern Med 126: 939–945. 9182470 10.7326/0003-4819-126-12-199706150-00002

[7] Reynolds SJ, Nakigozi G, Newell K, Ndyanabo A, Galiwongo R, et al (2009) Failiure of immunologic criteria to appropriately identify antiretroviral treatment failure in Uganda. AIDS 23: 697–700. 19209067 10.1097/QAD.0b013e3283262a78 PMC2720562

[8] World Health Organization (2010) Antiretroviral Therapy for HIV Infection in Infants and Children: Towards Universal Access: recommendations for a public health approach- 2010 Revision. Geneva: World Health Organization. 23741772

[9] Crowe SM, Turnbull SP, Oelrichs R, Dunne AL (2003) Monitoring Human Immunodeficiency Virus Infection in Resource-Constrained Countries. Clin Infect Dis Suppl 1: S25–S35. 12822129 10.1086/375369

[10] Fiscus SA, Cheng B, Crowe SM, Demeter L, Jennings C, et al (2006) HIV-1 Viral Load Assays for Resource-Limited Settings. PLos Med 3 10. 10.1371/journal.pmed.0030417 PMC1592347 17032062

[11] World Health Organization (2013) Consolidated guidelines on the use of antiretroviral drugs for treatment and preventing HIV infection. 24716260

[12] UNITAID (2012) HIV/AIDS Diagnostics Technology Landscape: Semi-Annual Update. Geneva: UNITAID.

[13] University of York (2008) Systematic Reviews: CRD's guidance for undertaking reviews in health care. York: Centre for Reviews and Dissemination.

[14] Bossuyt PM, Reitsma JB, Bruns DE, Gatsonis CA, Glasziou PP, et al (2003) Towards Complete and Accurate Reporting of Studies of Diagnostic Accuracy: The STARD Initiative. Clin Chem 49 1: 1–6. 12507953 10.1373/49.1.1

[15] Bossuyt PM, Reitsma JB, Bruns DE, Gatsonis CA, Glasziou PP, et al (2003) The STARD Statement for Reporting Studies of Diagnostic Accuracy: Explanation and Elaboration. Clin Chem 49 1: 7–18. 12507954 10.1373/49.1.7

[16] Bland MJ, Altman DG (1986) Statistical Methods for Assessing Agreement Between Two Methods of Clinical Measurement. Lancet 1: 307–310. 2868172

[17] Hughes MD, Johnson VA, Hirsch MS, Bremer JW, Elbeik T, et al (1997) Monitoring Plasma HIV-1 RNA Levels in Addition to CD4+ Lymphocyte Count Improves Assessment of Antiretroviral Therapeutic Response. Ann Intern Med 126 12: 929–938. 9182469 10.7326/0003-4819-126-12-199706150-00001

[18] Saag MS, Holodniy M, Kuritzkes DR, O'Brien WA, Coombs R, et al (1996) HIV viral load markers in clinical practice. Nat Med 2 6: 625–629. 8640545 10.1038/nm0696-625

[19] Braun P, Ehret R, Wiesmann F, Zabbai F, Knickmann M, et al (2007) Comparison of four commercial quantitative HIV-1 assays for viral load monitoring in clinical daily routine. Clin Chem Lab Med 45 1: 93–99. 17243923 10.1515/CCLM.2007.008

[20] Choi J-Y, Kim E-J, Rho HJ, Kim JY, Kwon O-K, et al (2009) Evaluation of the NucliSens EasyQ HIV-1 v1.1 and RealTime HIV-1 kits for quantitation of HIV-1 RNA in plasma. J Virol Methods 161 1: 7–11. 19576640 10.1016/j.jviromet.2009.02.003

[21] Church D, Gregson D, Lloyd T, Klein M, Beckthold B, et al (2011) Comparison of the realtime HIV-1, COBAS TaqMan 48 v1.0, easy Q v1.2, and Versant v3.0 assays for determination of HIV-1 viral loads in a cohort of Canadian patients with diverse HIV subtype infections. J of Clin Microbiol 49 1: 118–124. 21084515 10.1128/JCM.00685-10 PMC3020439

[22] Clarke JR, Galpin S, Braganza R, Ashraf A, Russell R, et al (2000) Comparative Quantification of Diverse Serotypes of HIV-1 in Plasma From a Diverse Population of Patients. J Med Virol 62: 445–449. 11074472 10.1002/1096-9071(200012)62:4<445::aid-jmv8>3.0.co;2-n

[23] Crump JA, Scott LE, Msuya E, Morrissey AB, Kimaro EE, et al (2009) Evaluation of the Abbott m2000rt RealTime HIV-1 assay with manual sample preparation compared with the ROCHE COBAS AmpliPrep/AMPLICOR HIV-1 MONITOR v1.5 using specimens from East Africa. J Virol Methods 162 1–2: 218–222. 19729037 10.1016/j.jviromet.2009.08.013 PMC2761523

[24] Damond F, Avettand-Fenoel V, Collin G, Roquebert B, Plantier JC, et al (2010) Evaluation of an upgraded version of the Roche Cobas AmpliPrep/Cobas TaqMan HIV-1 test for HIV-1 load quantification. J Clin Microbiol 48 4: 1413–1416. 20129964 10.1128/JCM.01409-09 PMC2849598

[25] Do T, Duncan J, Butcher A, Liegler T (2011) Comparative frequencies of HIV low-level viremia between real-time viral load assays at clinically relevant thresholds:. J Clin Virol 52 SUPPL. 1: S83–S89. 21995930 10.1016/j.jcv.2011.09.022

[26] Elbeik T, Alvord WG, Trichavaroj R, de Souza M, Dewar R, et al (2002) Comparative analysis of HIV-1 viral load assays on subtype quantification: Bayer Versant HIV-1 RNA 3.0 versus Roche Amplicor HIV-1 Monitor version 1.5. J Acquir Immune Defic Syndr 29 4: 330–9. 11917236 10.1097/00126334-200204010-00002

[27] Galli R, Merrick L, Friesenhahn M, Ziermann R (2005) Comprehensive comparison of the VERSANT HIV-1 RNA 3.0 (bDNA) and COBAS AMPLICOR HIV-1 MONITOR 1.5 assays on 1,000 clinical specimens. J Clin Virol 34 4: 245–252. 16286047 10.1016/j.jcv.2004.12.012

[28] Greengrass VL, Plate MM, Steele PM, Denholm JT, Cherry CL, et al (2009) Evaluation of the Cavidid ExaVir Load Assay (Version 3) for Plasma Human Immunodeficiency Virus Type 1 Load Monitoring. J Clin Microbiol 47 9: 3011–3013. 19605583 10.1128/JCM.00805-09 PMC2738117

[29] Gueudin M, Plantier JC, Lemee V, Schmitt MP, Chartier L, et al (2007) Evaluation of the Roche Cobas TaqMan and Abbott RealTime Extraction-Quantification Systems for HIV-1 Subtypes. J Acquir Immune Defic Syndr 44 5: 500–505. 17259908 10.1097/QAI.0b013e31803260df

[30] Karasi JC, Dziezuk F, Quennery L, Forster S, Reischl U, et al (2011) High correlation between the Roche COBAS AmpliPrep/COBAS TaqMan HIV-1, v2.0 and the Abbott m2000 RealTime HIV-1 assays for quantification of viral load in HIV-1 B and non-B subtypes. J Clin Virol 52 3: 181–186. 21813320 10.1016/j.jcv.2011.07.002

[31] Katsoulidou A, Rokka C, Issaris C, Haida C, Tzannis K, et al (2011) Comparative evaluation of the performance of the Abbott RealTime HIV-1 assay for measurement of HIV-1 plasma viral load on genetically diverse samples from Greece. Virol J 8 10. 10.1186/1743-422X-8-10 PMC3032708 21219667

[32] Labbett W, Garcia-Diaz A, Fox Z, Clewley GS, Fernandez T, et al (2009) Comparative evaluation of the ExaVir Load version 3 reverse transcriptase assay for measurement of human immunodeficiency virus type 1 plasma load. J Clin Mibrobiol 47 10: 3266–3270. 10.1128/JCM.00715-09 PMC2756939 19656978

[33] Muyldermans G, Debaisieux L, Fransen K, Marissens D, Miller K, et al (2000) Blinded, multicenter quality control study for the quantification of human immunodeficiency virus type 1 RNA in plasma by the Belgian AIDS reference laboratories. Clin Microbiol Infect 6 4: 213–217. 11168110 10.1046/j.1469-0691.2000.00048.x

[34] Oliver AR, Pereira SF, Clark DA (2007) Comparative Evaluation of the Automated Roche TaqMan Real-Time Quantitative Human Immunodeficiency Virus Type 1 RNA PCR Assay and the Roche AMPLICOR Version 1.5 Conventional PCR Assay. J Clin Microbiol 45 11: 3616–3619. 17804650 10.1128/JCM.00221-07 PMC2168503

[35] Paba P, Fabeni L, Ciccozzi M, Perno CF, Ciotti M (2011) Performance evaluation of the COBAS/TaqMan HIV-1 v2.0 in HIV-1 positive patients with low viral load: a comparative study. J Virol Methods 173 2: 399–402. 21419171 10.1016/j.jviromet.2011.03.014

[36] Pas S, Rossen JWA, Schoener D, Thamke D, Pettersson A, et al (2010) Performance evaluation of the new Roche Cobas AmpliPrep/Cobas TaqMan HIV-1 test version 2.0 for quantification of human immunodeficiency virus type 1 RNA. J Clin Microbiol 48 4: 1195–200. 20164281 10.1128/JCM.01832-09 PMC2849552

[37] Poveda E, de Mendoza C, Cuesta M, Toro C, Rodes B, et al (2003) Can drug resistance mutations influence the measurement of plasma HIV-RNA by different viral load techniques? AIDS Patient Care STDS 17 7: 321–324. 12952733 10.1089/108729103322231259

[38] Pyne MT, Konnick EQ, Phansalkar A, Hillyard DR (2009) Evaluation of the Abbott investigational use only realtime HIV-1 assay and comparison to the Roche Amplicor HIV-1 monitor test, version 1.5. J Mol Diagn 11 4: 347–354. 19460935 10.2353/jmoldx.2009.080166 PMC2710712

[39] Ruelle J, Jnaoui K, Lefevre I, Lamarti N, Goubau N (2009) Comparative evaluation of the VERSANT HIV-1 RNA 1.0 kinetic PCR molecular system (kPCR) for the quantification of HIV-1 plasma viral load. J Clin Virol 44 4: 297–301. 19230753 10.1016/j.jcv.2009.01.004

[40] Schutten M, Fries E, Burghoorn-Maas C, Niesters HGM (2007) Evaluation of the analytical performance of the new Abbott RealTime RT-PCRs for the quantitative detection of HCV and HIV-1 RNA. J Clin Virol 40 2: 99–104. 17768084 10.1016/j.jcv.2007.07.013

[41] Scott LE, Noble LD, Moloi J, Erasmus L, Venter WDF, et al (2009) Evaluation of the Abbott m2000 RealTime human immunodeficiency virus type 1 (HIV-1) assay for HIV load monitoring in South Africa compared to the Roche Cobas AmpliPrep-Cobas Amplicor, Roche Cobas AmpliPrep-Cobas TaqMan HIV-1, and BioMerieux NucliSENS EasyQ HIV-1 assays. J Clin Microbiol 47 7: 2209–2217. 19420172 10.1128/JCM.01761-08 PMC2708481

[42] Sire J-M, Vray M, Merzouk M, Plantier J-C, Pavie J, et al (2011) Comparative RNA quantification of HIV-1 group M and non-M with the Roche Cobas AmpliPrep/Cobas TaqMan HIV-1 v2.0 and Abbott Real-Time HIV-1 PCR assays. J Acquir Immune Defic Syndr 56 3: 239–243. 21164353 10.1097/QAI.0b013e3182099891

[43] Sivapalasingam S, Wangechi B, Marshed F, Laverty M, Essajee S, et al (2009) Monitoring virologic responses to antiretroviral therapy in HIV-infected adults in Kenya: Evaluation of a low-cost viral load assay. PLoS ONE 4 8. 10.1371/journal.pone.0006828 PMC2730572 19714253

[44] Sizmann D, Glaubitz J, Simon CO, Goedel S, Buergisser P, Drogan D, et al (2010) Improved HIV-1 RNA quantitation by COBAS AmpliPrep/COBAS TaqMan HIV-1 Test, v2.0 using a novel dual-target approach. J Clin Virol 49 1: 41–46. 20637687 10.1016/j.jcv.2010.06.004

[45] Sloma CR, Germer JJ, Gerads TM, Mandrekar JN, Mitchell PS, et al (2009) Comparison of the Abbott realtime human immunodeficiency virus type 1 (HIV-1) assay to the Cobas AmpliPrep/Cobas TaqMan HIV-1 test: workflow, reliability, and direct costs. J Clin Microbiol 47 4: 889–895. 19193837 10.1128/JCM.02231-08 PMC2668302

[46] Ssebugenyi I, Kizza A, Mpoza B, Aluma G, Boaz I, et al (2011) Comparison of the Abbott m2000 HIV-1 real-time and Roche AMPLICOR monitor v1.5 HIV-1 assays on plasma specimens from Rakai, Uganda. Int J STD AIDS 22 7: 373–375. 21729954 10.1258/ijsa.2009.009526 PMC3725460

[47] Stevens W, Horsfield P, Scott LE (2007) Evaluation of the Performance of the Automated NucliSENS easyMAG and EasyQ Systems versus the Roche AmpliPrep-AMPLICOR Combination for High-Throughput Monitoring of Human Immunodeficiency Virus Load. J Clin Microbiol 45 4: 1244–1249. 17267632 10.1128/JCM.01540-06 PMC1865857

[48] Swanson P, Huang S, Abravaya K, de Mendoza C, Soriano V, et al (2007) Evaluation of performance across the dynamic range of the Abbott RealTime HIV-1 assay as compared to VERSANT HIV-1 RNA 3.0 and AMPLICOR HIV-1 MONITOR v1.5 using serial dilutions of 39 group M and O viruses. J Virol Methods 141 1: 49–57. 17184853 10.1016/j.jviromet.2006.11.026

[49] Tang N, Huang S, Salituro J, Mak W-B, Cloherty G, et al (2007) A RealTi*me* HIV-1 viral load assay for automated quantitation of HIV-1 RNA in genetically diverse group M subtypes A-H, group O and group N samples. J Virol Methods 146: 236–245. 17707519 10.1016/j.jviromet.2007.07.003

[50] Troppan KT, Stelzl E, Violan D, Winkler M, Kessler HH (2009) Evaluation of the new VERSANT HIV-1 RNA 1.0 Assay (kPCR) for quantitative detection of human immunodeficiency virus type 1 RNA. J Clin Virol 46 1: 69–74. 19576845 10.1016/j.jcv.2009.06.012

[51] van Rensburg EJ, Tait K, Watt A, Schall R (2011) Comparative evaluation of the Roche Cobas AmpliPrep/Cobas TaqMan HIV-1 version 2 test using the TaqMan 48 analyzer and the Abbott RealTime HIV-1 assay. J Clin Microbiol 49 1: 377–379. 20980564 10.1128/JCM.01285-10 PMC3020483

[52] Weissman S, Bekui A, Darbhanga B, Ngo N, Amico C (2009) HIV Viral Load Monitoring: The Clinical Impact of Changing from RT-PCR to bDNA. J Clin Outcomes Manage 16 2: 75–80.

[53] Wirden M, Tubiana R, Marguet F, Leroy I, Simon A, et al (2009) Impact of Discrepancies between the Abbott RealTime and Cobas TaqMan Assays for Quantification of Human Immunodeficiency Virus TYpe 1 Group M Non-B Subtypes. J Clin Microbiol 47 5: 1543–1545. 19297599 10.1128/JCM.02134-08 PMC2681834

[54] Xu S, Song A, Nie J, Li X, Meng S, et al (2012) Comparison between the automated Roche Cobas AmpliPrep/Cobas TaqMan HIV-1 test version 2.0 assay and its version 1 and Nuclisens HIV-1 EasyQ version 2.0 assays when measuring diverse HIV-1 genotypes in China. J Clin Virol 53 1: 33–37. 22051503 10.1016/j.jcv.2011.10.001

[55] Young TP, Cloherty G, Fransen S, Napolitano L, Swanson P, et al (2011) Performance of the Abbott RealTime HIV-1 viral load assay is not impacted by integrase inhibitor resistance-associated mutations. J Clin Microbiol 49 4: 1631–1634. 21289145 10.1128/JCM.02253-10 PMC3122809

[56] Stevens W, Gelman R, Glencross DK, Scott LE, Crowe SM, et al (2008) Evaluating new CD4 enumeration technologies for resource-constrained countries. Nat Rev Microbiol Supplement S29–S38. 10.1038/nrmicro2000 22745957

