# Supplementary figures and images for: Systematic Review of the Performance of HIV Viral Load Technologies on Plasma Samples
Source: PLoS One. 2014 Feb 18;9(2):e85869. doi: 10.1371/journal.pone.0085869 (PMC3928047; doi:10.1371/journal.pone.0085869)

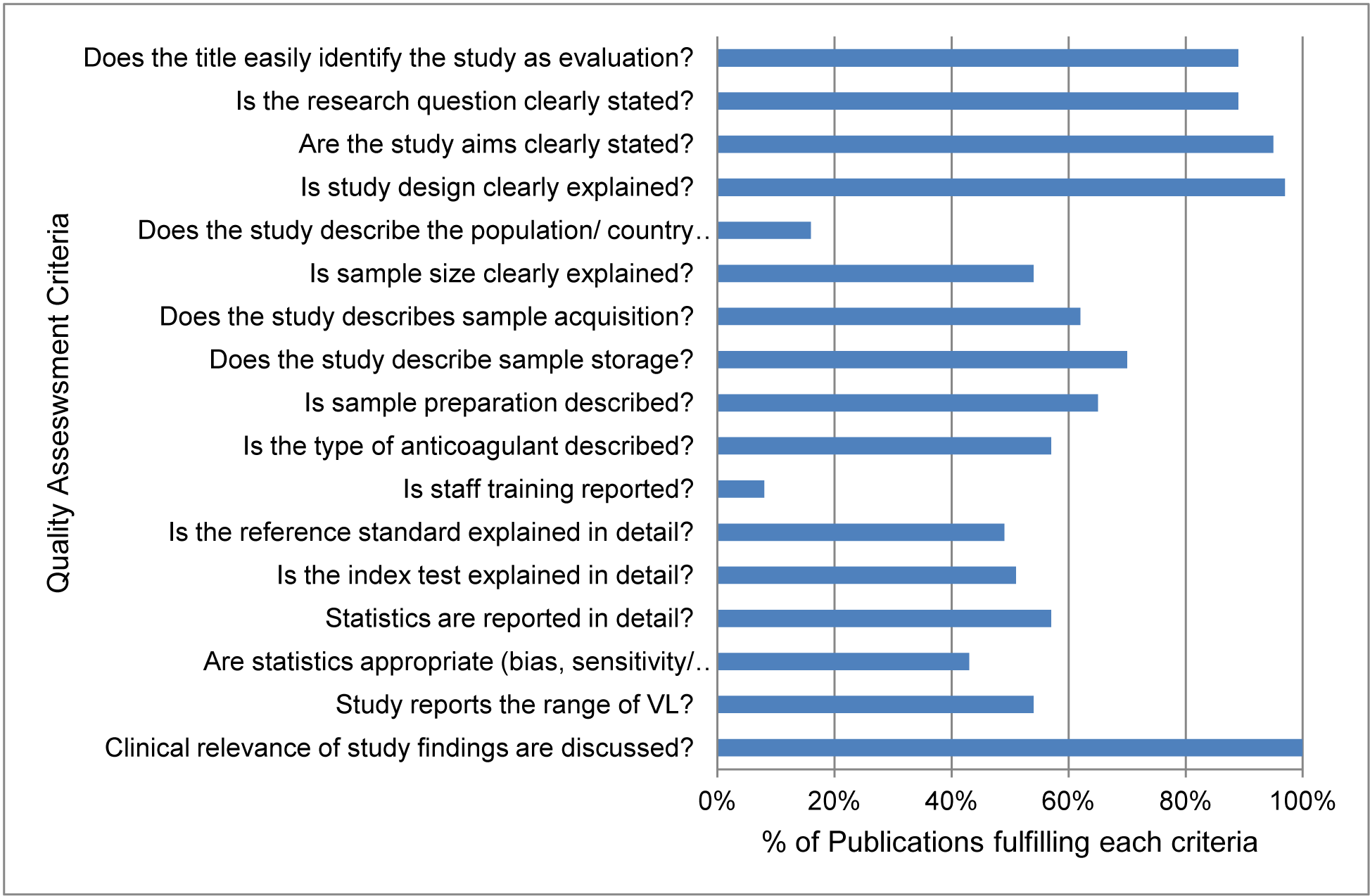

Supplement: Annex S1 — Quality assessment results by 17 criteria. (TIF) [file pone.0085869.s001.tif]
