# Supplementary material for: Systematic Review of the Performance of HIV Viral Load Technologies on Plasma Samples
Source: PLoS One. 2014 Feb 18;9(2):e85869. doi: 10.1371/journal.pone.0085869 (PMC3928047; doi:10.1371/journal.pone.0085869)
Supplement: Annex S2 — Review Protocol. (DOCX) [file pone.0085869.s002.docx]

**Systematic Review of Viral Load Diagnostic Assay**

**Review Team**

Kimberly Sollis, Pieter Smit, Rosanna Peeling

**Advisory Group**

David Barnett, Ben Cheng, Suzanne Crowe, Tom Denny, Susan Fiscus, Alan Landay, Thomas Spira, Wendy Stevens

**Background**

In an HIV-infected individual, the concentration of virus in the bloodstream or *viral load* (VL) can be a valuable tool for the clinical management of the infection. Broadly, there are three clinical uses for quantifying HIV in plasma: diagnosing acute HIV infection; determining prognosis and risk of opportunistic infection; and therapeutic monitoring. Unlike antibody detection, which is confounded by the trans-placental transfer of maternal IgG molecules, VL can also be useful in diagnosing babies born to HIV-positive mothers. Monitoring VL is most relevant worldwide for its third use, as a biomarker to evaluate the therapeutic efficacy of ART.

Antiretroviral therapy (ART) interrupts viral replication decreasing the levels of virus in the bloodstream thereby slowing the progression to AIDS and improving the prognosis. Initiation of therapy is determined by the patient’s CD4 T-cell count; however, once therapy has commenced, VL is used as an indicator of therapeutic success. The current aim of antiretroviral therapy should be to maintain the VL below the level of detection of available assays. Upon initiation of treatment, VL reduction indicates treatment efficacy correlating with an improved clinical outcome. VL is increased in individuals on anti-retroviral therapy if there is a low drug concentration in the body because of non-adherence or poor pharmacokinetics, and in the emergence of drug resistance. Other factors that can increase VL include acute infection and immunizations; however, these increases are modest and transient.

Commercially available VL assays differ in their sensitivity, dynamic range, target region and methods of nucleic acid detection. The differences in the region targeted by the assays means that they also differ in their versatility and ability to detect and quantify different HIV tropisms.

HIV-1 is responsible for the majority of infections around the world and is divided into three groups (M, N, O), and 9 subtypes and two sub-subtypes within the M group (A1, A2, B, C, D, F1, F2, G, H, J, K). There are also 14 circulating recombinant forms (CRF) or inter-subtype recombinant HIV-1 (CRF01_AE to CRF14_BG). A study in 2004 analysing the global distribution of HIV-1subtypes illustrated the relative contribution of each of the subtypes and recombinants to the overall burden of HIV-1 infection (Table 1) (Hemelaar et al, 2006).

**Table 1. Subtypes and recombinants responsible for HIV-1 infections in 2004.**

| **HIV-1 Subtype** | **(%) of 2004 HIV-1 positive patients infected** | **HIV-1 Recombinant** | **(%) of 2004 HIV-1 positive patients infected** |
| --- | --- | --- | --- |
| C | 50% | CRF01_AE | 5% |
| A | 12% | CRF02_AG | 5% |
| B | 10% | CRF03_AB | 0.1% |
| D | 3% | Other Recombinants | 8% |
| G | 6% | All Recombinants | 18% |
| F, H, J, K | 0.94% |  |  |

HIV-2 is much less virulent and transmissible than HIV-1 and is the cause of a small minority of HIV cases in Western and Central Africa.

The prevalence of HIV-1 and HIV-2 groups and subtypes differ across geographical regions and the choice of diagnostic assays should reflect the geographic distribution of the groups and subtypes. Versatility is also an important factor given the movement of subtypes and emergence of unidentified recombinant forms. For example, subtype B is the main cause of the HIV epidemic in Western Europe and North America but non-B strains and CRFs have been identified in HIV-1 positive patients. VL assays should therefore detect and quantify all known HIV-1 subtypes, inter-subtype recombinants and emerging variants to provide the greatest benefit.

At present, there are no international regulations or governing body to monitor commercially available diagnostic tests. Countries depend on the information published on manufacturer’s websites to guide decision-making and the formulation of national policies and guidelines. VL is a particularly important aspect of HIV infection to monitor because of the virus’s ability to mutate and cause treatment failure. Quantifying VL enables a clinician to assess the success of treatment and observe treatment failure. This facilitates an evidence- based approach to patient management and can improve the specificity of treatment (WHO A guide for diagnostic evaluations). A systematic-review would inform policy makers of the characteristics of diagnostic assays and facilitate evidence-based decision making. This will be accomplished through a systematic review of evaluations of VL assays published in English, in peer-reviewed journals.

**Review Objectives**

**Primary Objective**

To assess the test performance of commercially available VL assays.

**Secondary Objective**

To assess the operational characteristics of commercially available VL assays.

**PICOS**

**Participants**

Studies of performance of VL enumeration (quantification) technologies in HIV positive adults, in any geographical location.

**Interventions (Diagnostic Assays)**

Any commercially available technology available for the quantification of plasma HIV-1 VL, including signal and target amplification and detection technology.

**Reference standard/ comparators**

Reverse-transcriptase PCR

**Outcomes**

Include studies evaluating subtype quantitation and linear range.

Include comparative studies evaluating subtype quantitation and linear range.

**Study Design**

Include evaluative studies using an acceptable reference technology and comparative studies.

**Identifying research evidence**

**Search strategy**

Databases: Embase, Global Health/ Global Health Archive, Medline, Web of Science, Africa-Wide NiPAD, LILACS

Manufacturers’ websites and contact with manufacturers

MEDLINE: All aspects of clinical medicine, biomedicine, nursing, dentistry, allied health, health policy, genetics ect. Emphasis on English-language source, quite biased towards journals published in N. America. Includes journal articles from 1950s onwards and is updated daily.

EMBASE: All aspects of clinical medicine, biomedicine, nursing, dentistry, allied health, health policy, genetics ect. Quite biased towards information in journals published in Europe. Particularly strong on pharmaceutical information. Includes journals from 1947 onwards and is updated weekly.

**Search Terms**

Search terms to include:

HIV-1, HIV-2, HIV, human immunodeficiency virus type-1, human immunodeficiency virus type-2, human immunodeficiency virus

Viral load, RNA, ribonucleic acid, RT, reverse transcriptase

Assay, analysis, assess*, compar*, detection, eval*, monitor, measure*, quantify*, quantitation, reproducibility, technology, test, var*

Accuracy, correlation, performance, precision, reliability, sensitivity, specificity

Amplicor HIV-1 Monitor v1.5 (Roche Diagnostics)

COBAS Amplicor HIV-1 Monitor v1.5 (Roche Diagnostics)

COBAS AmpliPrep/COBAS Amplicor HIV-1 Monitor v1.5 (Roche Diagnostics)

COBAS AmpliPrep/COBAS TaqMan HIV-1 Test versions 1 and 2 (Roche Diagnostics)

Versant HIV-1 RNA 3.0 (Siemens)

NucliSens EasyQ HIV-1 v1.1 (bioMerieux)

RealTime HIV-1 Assay (Abott Laboratories)

ExaVir Load (Cavidi AB)

Versant k-PCR Molecular Systems (Siemens)

**Date**

1990 to present

**Language**

English

**Publication type/status**

Published works in peer reviewed journals

**Study selection**

Stage 1: Screening of titles/ abstracts against inclusion criteria.

Titles and abstracts, where available, will be screened and either accepted, rejected as not relevant, or rejected due to failure to meet inclusion criteria (if so, the reason will be specified).

Stage 2: Full papers obtained and assessed against inclusion criteria. Papers will be either accepted or rejected due to failure to meet inclusion criteria and the reason will be specified.

Full papers will be independently assessed by both members of the review team and results will be compared. Discrepancies will be resolved by consensus following a review of the protocol.

**Inclusion Criteria**

- Evaluation or comparison of performance of commercially available VL quantification assays
- Any nucleic acid extraction, amplification, detection method, and any target region
- Linear range (RNA copies/mL) of 40 to 10,000,000
- Any HIV-1 or HIV-2 subgroup recognition
- Quantitation of *plasma* VL

**Algorithm for Inclusion**

**
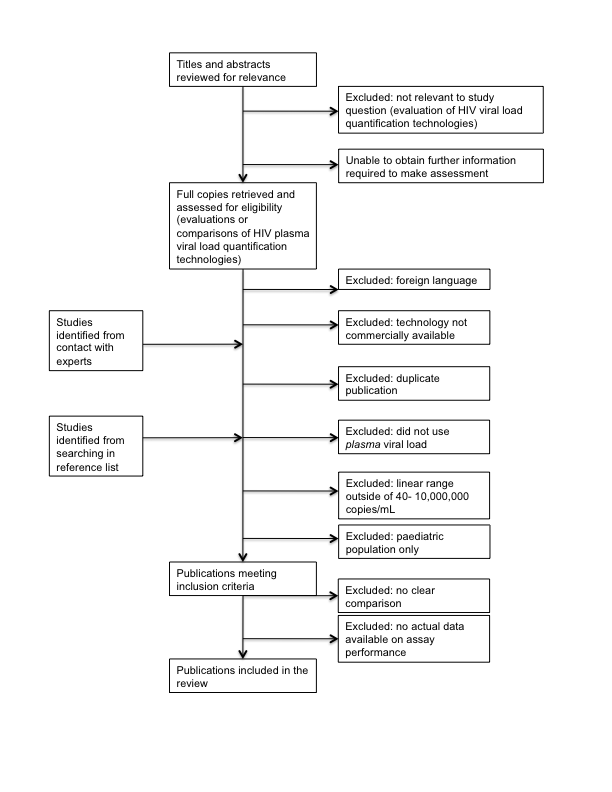
**

**Data Extraction**

| **General Information** |  |
| --- | --- |
| Researcher performing data extraction |  |
| Date of data extraction |  |
| Identification features of the study:  Record number  Author  Article title  Citation Type of publication  Country of origin  Source of funding |  |
|  |  |
| **Study Characteristics** |  |
| Aim/ objectives of the study |  |
| Study design |  |
| Study inclusion and exclusion criteria |  |
| Blinding of technicians |  |
|  |  |
| **Participant/ Sample Characteristics** |  |
| Characteristics of population from which samples were drawn:  Age  Sex  HIV +/-  ART +/- |  |
| Number of samples |  |
| Sample country/ region of origin |  |
| How were the samples acquired? |  |
| Sample type |  |
| HIV-1 subtype |  |
| Sample storage |  |
| Anticoagulant used |  |
| Method of sample preparation |  |
|  |  |
| **Technology** |  |
| Name and manufacturer of VL quantification assays under evaluation |  |
| Nucleic Acid Extraction method |  |
| Amplification method |  |
| Detection method |  |
| Target region |  |
| Reference standard (if any) |  |
|  |  |
| **Outcome data/ results** |  |
| Unit of assessment/ analysis |  |
| Statistical techniques used |  |
| Outcomes:  Mean VL + standard deviation  Correlation and agreement  Linear range  Sensitivity  Specificity  Accuracy/ precision  Predictive Value |  |
| For each pre-specified outcome:  Reported (Y/N)  Definition used in study  Unit of measurement |  |
| Additional outcomes reported |  |
| Details of any additional relevant outcomes reported |  |

**Quality Assessment**

| **Title/ abstract** | Is the article easily identified as a study of test evaluation? |  |
| --- | --- | --- |
| **Introduction** | Does it clearly state the research question and study aims? |  |
| **Methods** | Do the authors clearly describe the study design? |  |
|  | Are study inclusion and exclusion criteria provided? |  |
|  | Were steps taken to introduce blinding and random allocation where possible and appropriate? |  |
| Participant/ Sample Characteristics | Is the population from which the samples were drawn described? |  |
|  | Is the country/ region of sample origin detailed? |  |
|  | Were the samples collected prospectively? |  |
|  | Do the authors describe how the samples were acquired, stored and prepared? |  |
|  | Was the choice of anticoagulant appropriate for the technology? |  |
| Test methods | Were staff trained in the use of the technology prior to performing the index and reference tests? |  |
|  | Was the reference standard explained in sufficient detail to be reproduced? |  |
|  | Is the index test explained in sufficient detail to be reproduced? |  |
|  | Did the authors report the number of technicians reading the index/ reference tests? |  |
|  | Were the technicians performing the index/ reference tests blinded to the result of the other method? |  |
|  | Was a single sample divided and tested by each technology included in the study or were a different set of samples used for each test? |  |
| Statistical methods | Were the methods used reported in detail? |  |
|  | Were the methods used appropriate? |  |
| **Results** |  |  |
| Participants | Were the demographic characteristics of the population described? |  |
| Test results | Was the distribution/ range of VL of the sampled population reported? |  |
|  | How was the data presented? |  |
|  | Was subgroup analysis performed for different HIV-1 subtypes? |  |
| **Discussion** | Was the clinical relevance of the study findings discussed? |  |

**Objective 2:** To fulfill the second objective of this systematic review, the following information will be collected from publications, contact with the manufacturers, and interviews with users:

| Name of commercially available VL assay |  |
| --- | --- |
| Manufacturing company |  |
|  |  |
| Nucleic Acid Extraction Method |  |
| Amplification Method |  |
| Detection Method |  |
| Target Region |  |
| Linear Range (RNA Copies/mL) |  |
| HIV-1 Subgroup Recognition |  |
| Specimen Compatibility (Anticoagulant) |  |
| Specimen Volume Requirements (mL) |  |
| Quantitation Standards (QS) |  |
|  |  |
| Steps required to perform the assay |  |
| Reagents used |  |
| Materials required but not supplied |  |
| Time to result |  |
| Throughput |  |
| Training required |  |
| Robustness to heat, humidity, etc |  |
| Cost of equipment |  |
| Cost per test |  |
| Maintenance |  |
| Availability of QA/QC reagents |  |
| Reproducibility |  |
